# Everolimus downregulates STAT3/HIF-1α/VEGF pathway to inhibit angiogenesis and lymphangiogenesis in *TP53* mutant head and neck squamous cell carcinoma (HNSCC)

**DOI:** 10.18632/oncotarget.28355

**Published:** 2023-02-02

**Authors:** Md Maksudul Alam, Janmaris Marin Fermin, Mark Knackstedt, Mackenzie J. Noonan, Taylor Powell, Landon Goodreau, Emily K. Daniel, Xiaohua Rong, Tara Moore-Medlin, Alok R. Khandelwal, Cherie-Ann O. Nathan

**Affiliations:** ^1^Department of Otolaryngology-Head and Neck Surgery, LSU-Health Sciences Center, Shreveport, LA 71103, USA; ^2^School of Medicine, LSU-Health Sciences Center, Shreveport, LA 71103, USA; ^3^Feist-Weiller Cancer Center, LSU-Health Sciences Center, Shreveport, LA 71103, USA

**Keywords:** TP53 mutant, HNSCC, angiogenesis, everolimus, mTOR

## Abstract

*TP53* mutant head and neck squamous cell carcinoma (HNSCC) patients exhibit poor clinical outcomes with 50–60% recurrence rates in advanced stage patients. In a recent phase II clinical trial, adjuvant therapy with everolimus (mTOR inhibitor) significantly increased 2-year progression-free survival in p53 mutated patients. TP53-driven mTOR activation in solid malignancies causes upregulation of HIF-1α and its target, downstream effector VEGF, by activating STAT3 cell signaling pathway. Here, we investigated the effects of everolimus on the STAT3/HIF-1α/VEGF pathway in *TP53* mutant cell lines and xenograft models. Treatment with everolimus significantly inhibited cell growth *in vitro* and effectively reduced the growth of *TP53* mutant xenografts in a minimal residual disease (MRD) model in nude mice. Everolimus treatment was associated with significant downregulation of STAT3/HIF-1α/VEGF pathway in both models. Further, treatment with everolimus was associated with attenuation in tumor angiogenesis and lymphangiogenesis as indicated by decreased microvessel density of vascular and lymphatic vessels in HN31 and FaDu xenografts. Everolimus downregulated the STAT3/HIF-1α/VEGF pathway to inhibit growth and *in vitro* tube formation of HMEC-1 (endothelial) and HMEC-1A (lymphatic endothelial) cell lines. Our studies demonstrated that everolimus inhibits the growth of *TP53* mutant tumors by inhibiting angiogenesis and lymphangiogenesis through the downregulation of STAT3/HIF-1α/VEGF signaling.

## INTRODUCTION

HNSCC ranks sixth amongst cancers diagnosed worldwide [1]. Despite recent advancements in treatment modalities, 50–60% of human papillomavirus (HPV) negative advanced stage HNSCC patients develop locoregional recurrence after definitive treatment [1, 2]. *TP53* is the most frequently mutated gene in ≥80% of HPV-negative tumors [3–5]. This is unlike p53 wild-type (wt) tumors seen more commonly in HPV-positive HNSCC which have excellent survival rates that now require de-escalation of treatment. In addition to losing tumor-suppressive functions, mutant p53 proteins acquire additional biological functions with transforming abilities that promote tumorigenesis [6, 7]. Mutant p53 causes sustained activation of the mTOR pathway, thereby contributing to cancer pathogenesis [8, 9]. Our prior work has shown that overexpression of eIF4E in surgical margins predicts recurrence and that overexpression of eIF4E is functionally active through activation of the Akt/mTOR pathway [10, 11]. Results from a window of opportunity clinical trial concluded that the mTOR pathway was a potential therapeutic target for HNSCC [12]. In our recently reported phase-2 multi-institutional adjuvant trial with everolimus in stage IV HNSCC patients at high risk for recurrence, treatment with everolimus demonstrated significant improvement in 2-year progression-free survival (PFS) in patients with *TP53* mutations compared to the placebo group [13]. Our results are even more exciting as this subset of patients with *TP53* mutations has the highest risk of recurrence and therefore, the greatest need for adjuvant therapy [14, 15]. Hence, this study focuses on p53 mutated tumors as it is this group of patients that requires adjuvant therapy to improve survival. HNSCC cells exhibit a significant upregulation in lymphatic endothelial cell invasiveness and proliferation [16, 17]. The role of mTOR inhibitors (mTORi) as potent growth inhibitory and antiangiogenic/anti-lymphangiogenic agents in HNSCC is well established [18]. Moreover, mTORi significantly suppressed baseline invasiveness of endothelial and HNSCC tumor cells [19]. However, the underlying molecular mechanisms for mutant p53 protein-mediated activation of the mTOR pathway which drive the oncologic processes in HNSCC are yet to be elucidated. Previous studies have shown that mutant p53 causes stabilization of HIF-1α to upregulate its transcriptional activity, promoting a variety of oncologic processes, including angiogenesis and lymphangiogenesis [20–22]. Moreover, mutant p53-mediated activation of HIF-1α is both transcriptionally and translationally regulated through mTOR [23, 24]. HIF-1α target genes, such as VEGF-A and VEGF-C, are involved in tumor angiogenesis and lymphangiogenesis [25–27], leading to lymph node metastasis and recurrence [28–30]. Accordingly, we sought to investigate the mechanism for everolimus-induced inhibition of TP53 HNSCC.

## RESULTS

### Everolimus inhibits the growth of TP53 mutant HNSCC both *in vitro* and *in vivo*

**Figure 1:**
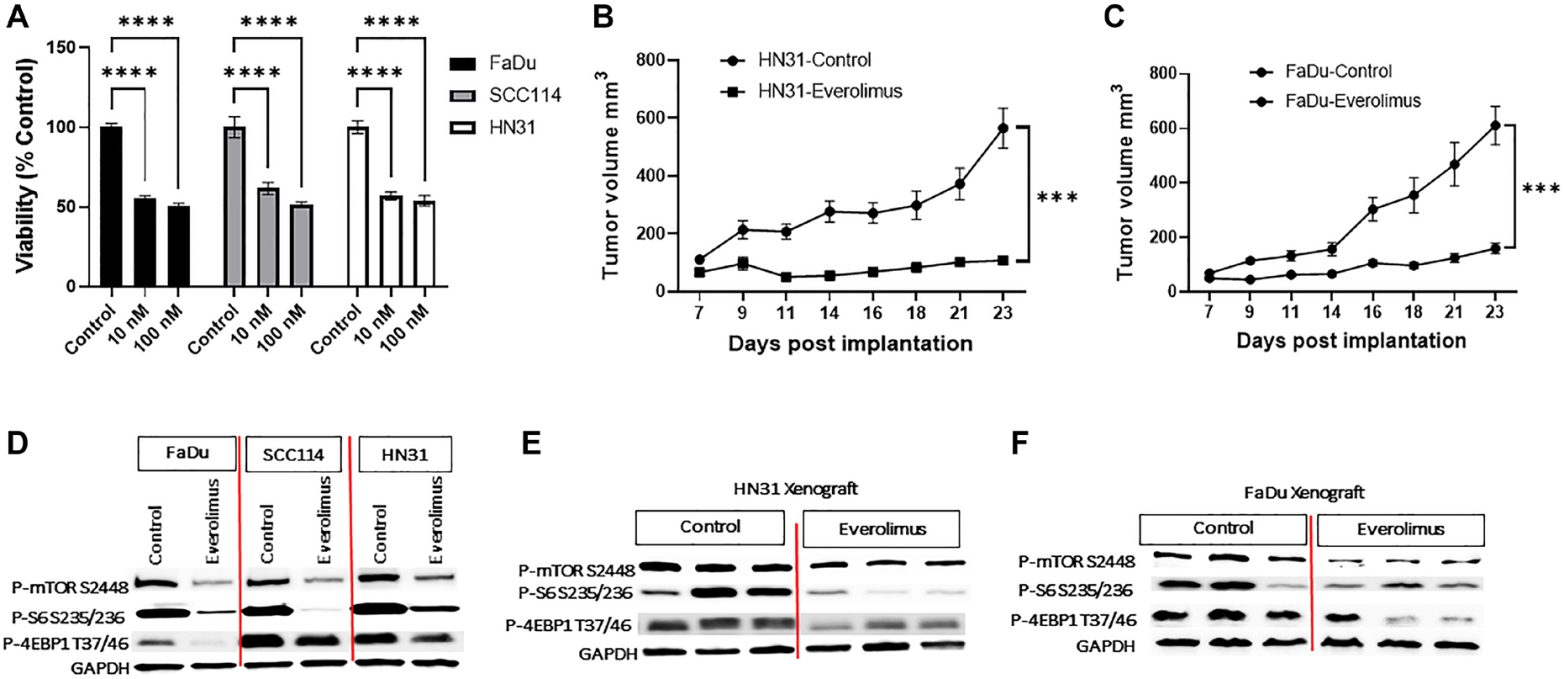
Everolimus downregulates mTORC1 pathway and inhibits growth of *TP53* mutant HNSCC cell lines and xenografts. (**A**) The effect of everolimus on cell viability was measured using an MTS assay after six days of treatment. Cell viability of everolimus treated cells was measured as a percent of control (untreated cells). ^****^ *P* < 0.00005 vs. control, ANOVA. Data represent the mean +/− SEM of three independent experiments, each experiment comprising samples in triplicate. (**B**) The growth curve of HN31 xenograft showing everolimus significantly reduces tumor volume. ^***^ *P* < 0.0005 vs. control, Student’s *t*-test. Data represent mean +/− SEM, *N* = 20 mice in each group. (**C**) The growth curve of FaDu xenograft showing that everolimus significantly reduces tumor volume. ^***^ *P* < 0.0005 vs. control, Student’s *t*-test. Data represent mean +/− SEM, *N* = 20 mice in each group. (**D**) Representative western blot for mTOR pathway proteins, prepared from cells treated with 100 nm everolimus for 24 hours. (**E** and **F**) Representative western blot for mTOR pathway proteins in HN31 and FaDu xenografts. The levels of P-mTOR, P-S6 and P-4EBP1 were reduced in everolimus treated cell lines and xenografts. The western blot experiment was repeated three times.

To evaluate the effect of everolimus on the cell growth of *TP53* mutant HNSCC, we utilized FaDu, SCC114, and HN31 cell lines. Consistent with our previous study, everolimus significantly reduced cell viability at 10 nm and 100 nm concentrations (Figure 1A). Moreover, we also evaluated the effect of everolimus on *TP53* mutant HNSCC tumor growth *in vivo* in a tumor mouse xenograft model. Treatment with everolimus (5 mg/kg, oral gavage daily) for 23 days significantly inhibited the growth of both HN31 (Figure 1B) and FaDu xenografts suggesting the inhibitory effect is not cell line specific (Figure 1C).

### Everolimus downregulates mTORC1 pathway in *TP53* mutant HNSCC

We next evaluated the effect of everolimus on the oncogenic mTOR signaling pathway. Treatment of FaDu, SCC114, and HN31 cells with everolimus significantly downregulated P-mTOR S2448, P-S6 S235/236, and P-4EBP1 T37/46 in these cell lines (Figure 1D). To further translate our *in vitro* studies *in vivo*, we investigated the effect of everolimus on the mTORC1 pathway in HN31 and FaDu tumor cell xenografts. Consistent with the effect of everolimus *in vitro*, oral administration of everolimus was associated with a significant downregulation of P-mTOR S2448, P-S6 S235/236, and P-4EBP1 T37/46 in HN31 and FaDu xenografts (Figure 1E and 1F).

### Everolimus inhibits STAT3 phosphorylation and downregulates HIF-1α, VEGF-A and VEGF-C in *TP53* mutant HNSCC

**Figure 2:**
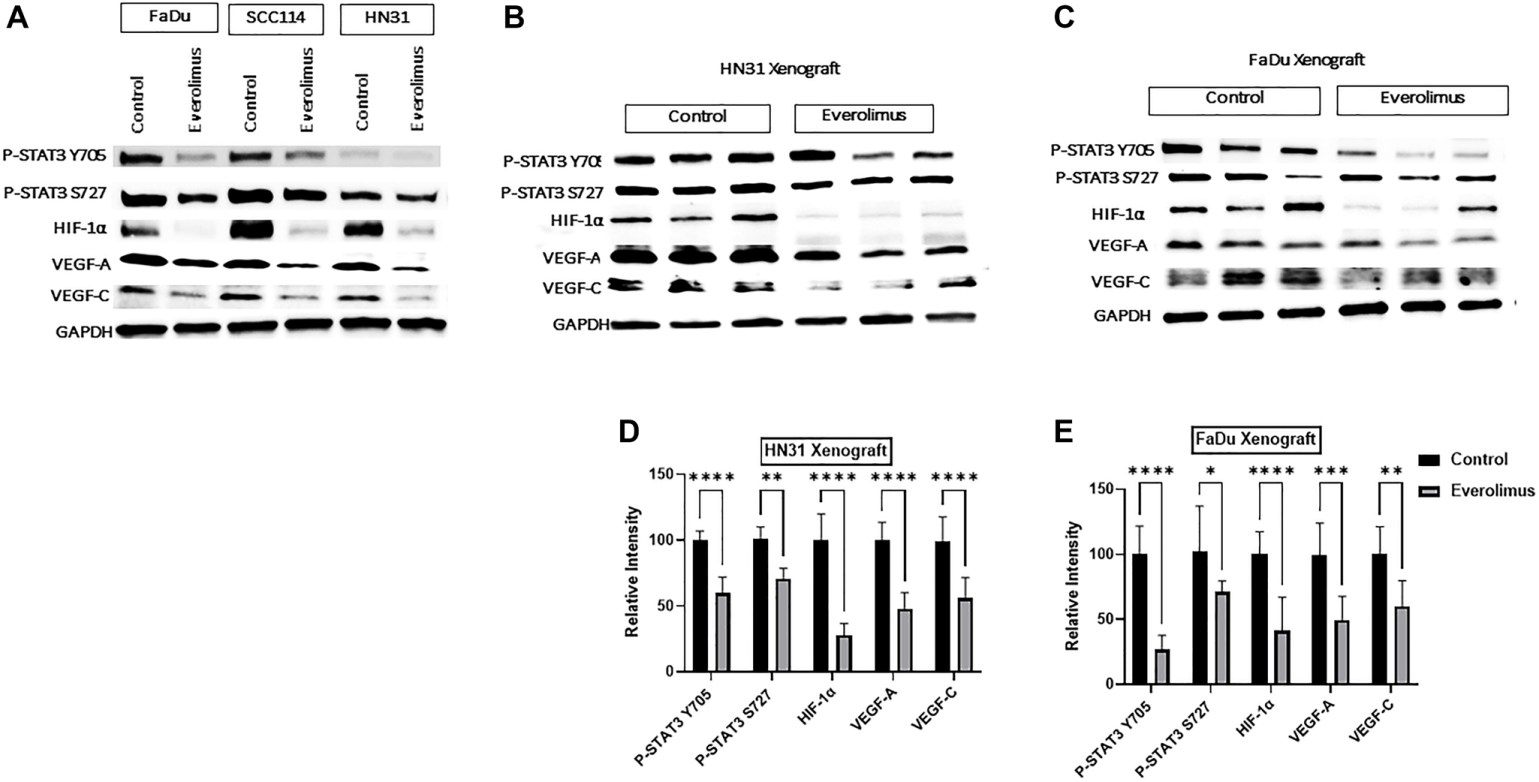
Everolimus inhibits STAT3 phosphorylation and downregulates HIF-1α, VEGF-A and VEGF-C in *TP53* mutant HNSCC cell lines and xenografts. (**A**) Representative western blot of P-STAT3, HIF-1α, VEGF-A and VEGF-C in FaDu, SCC114, and HN31 cell lines. Proteins were prepared from cells treated with 100 nm everolimus for 24 hours. (**B** and **C**) Representative western blot for P-STAT3, HIF-1α, VEGF-A, and VEGF-C in HN31 and FaDu xenograft. Proteins were isolated from the xenograft after mice were treated with 5 mg/kg of everolimus daily for 21 days. The western blot experiment was repeated three times. Everolimus also downregulated HIF-1α and its target genes VEGF-A and VEGF-C in FaDu, SCC114, and HN31 cell lines. This downregulation was also seen in both xenograft models (**B**–**E**). Moreover, the mRNA levels of HIF-1α, VEGF-A and VEGF-C were significantly reduced by everolimus treatment both *in vitro* (Figure 3A–3C) and *in vivo* (Figure 3D–3E). Secretion of VEGF-A was also reduced when cells were treated with everolimus (Figure 3F), as measured by ELISA.

**Figure 3:**
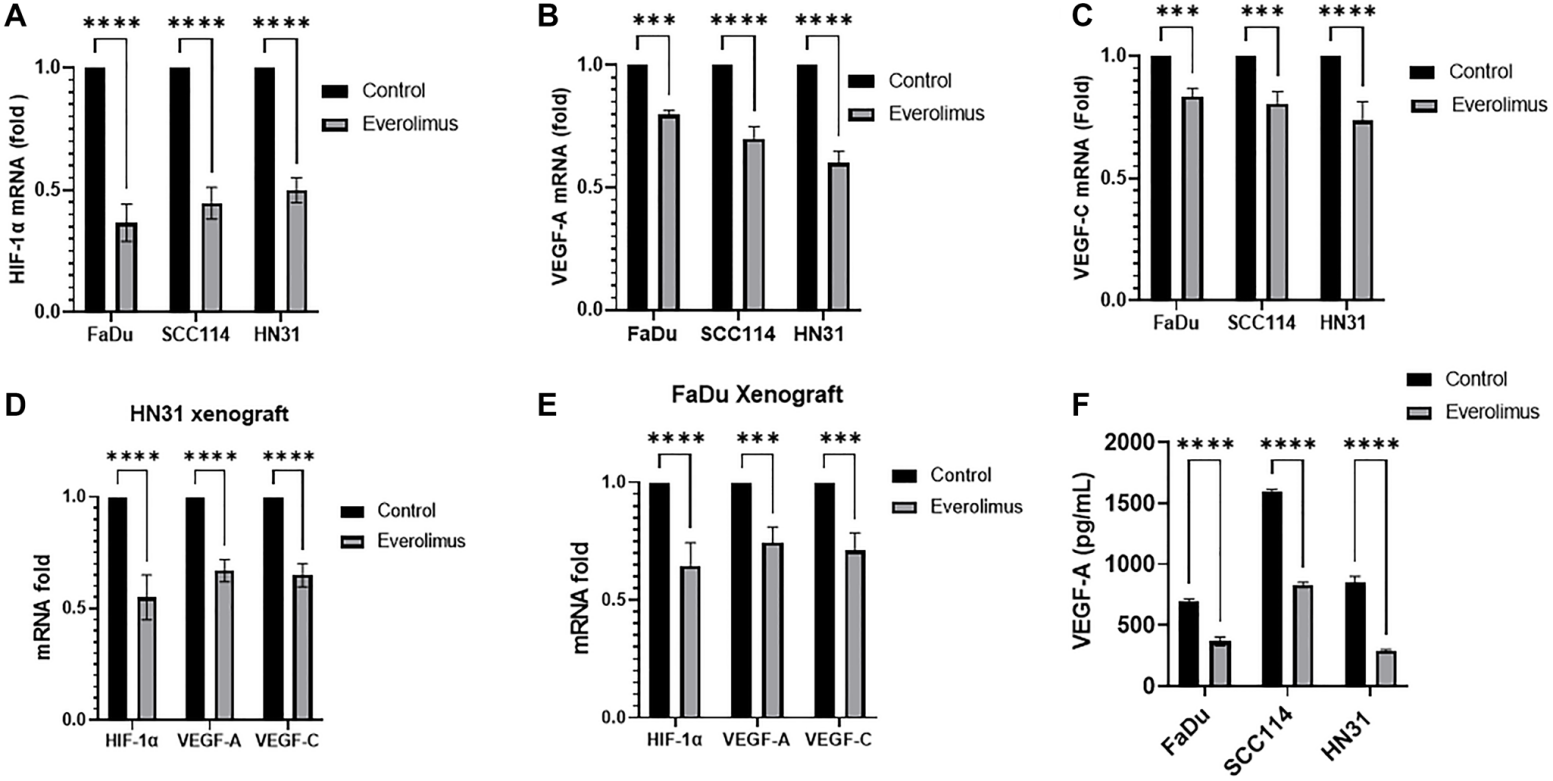
Everolimus reduces mRNA levels of HIF-1α, VEGF-A and VEGF-C in *TP53* mutant HNSCC cell lines and xenografts. qRT-PCR was employed to measure the relative amount of mRNA. (**A**–**C**) mRNA fold change for HIF-1α, VEGF-A and VEGF-C in FaDu, SCC114, and HN31 cell lines, fold changes are shown relative to control. ^**^ *P* < 0.005; ^***^ *P* < 0.0005; ^****^ *P* < 0.00005 vs. control, ANOVA. Data represent the mean +/− SEM of three independent experiments, each comprising samples in triplicate. (**D** and **E**) mRNA fold change for HIF-1α, VEGF-A and VEGF-C in HN31 and FaDu xenograft. ^***^ *P* < 0.0005; ^****^ *P* < 0.00005 vs. control, ANOVA. Data represent the mean +/− SEM of three independent experiments comprising *n* = 3 mice. (**F**) Everolimus reduces the secretion of VEGF-A in cell culture medium. The level of VEGF-A in cell culture medium was determined using ELISA. ^****^ *P* < 0.00005 vs. control, ANOVA. Data represent the mean +/− SEM of three independent experiments comprising triplicate samples in each experiment.

As the activity of the mTORC1 pathway is upstream of HIF-1α, we investigated whether everolimus can reduce the level of HIF-1α and its targets VEGF-A and VEGF-C both in cell lines and xenografts [31]. Western blot analysis demonstrated that everolimus reduced P-STAT3 Y705 and P-STAT3 S727 not only in cell lines *in vitro*, but also in tumor-cell xenografts (Figure 2A–2E). The levels of HIF-1α, VEGF-A and VEGF-C were also decreased by everolimus treatment in cell lines (Figure 2A) as well as in xenografts (Figure 2B–2E). Moreover, mRNA levels of HIF-1α, VEGF-A and VEGF-C were significantly reduced by everolimus treatment both *in vitro* (Figure 3A–3C) and *in vivo* (Figure 3D and 3E). Secretion of VEGF-A was also reduced when cells were treated with everolimus (Figure 3F), as measured by ELISA.

### Everolimus inhibits tumor angiogenesis and lymphangiogenesis in mouse xenograft model

**Figure 4:**
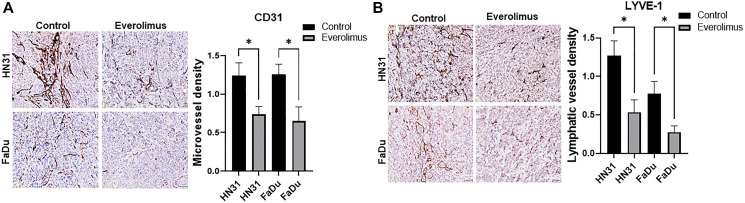
Everolimus reduces microvessel density (MVD) and lymphatic vessel density (LVD) in TP53 mutant HNSCC xenografts. (**A**) Representative image of CD31 immunostaining and quantification of MVD in HN31 and FaDU xenografts. (**B**) Representative image of LYVE-1 immunostaining and quantification of LVD in HN31 and FaDU xenografts. Prior to tumor harvesting, mice were treated with 5 mg/kg of everolimus daily for 21 days. ^*^ *P* < 0.05 vs. control, ANOVA. Data represent the mean +/− SEM, *N* = 20 mice in each group.

We investigated the effect of everolimus on tumor angiogenesis and lymphangiogenesis. CD31 immunohistochemistry demonstrated that everolimus significantly reduced microvessel density in HN31 and FaDu xenografts (Figure 4A). To identify tumor-associated lymphatic vessels, tumors were immunostained with mouse-specific LYVE-1. Consistent with the inhibitory effect of everolimus on angiogenesis, lymphatic vessel density was significantly reduced by everolimus treatment in FaDu and HN31 xenografts (Figure 4B).

### The effect of everolimus on human microvascular endothelial (HMEC-1) cells

**Figure 5:**
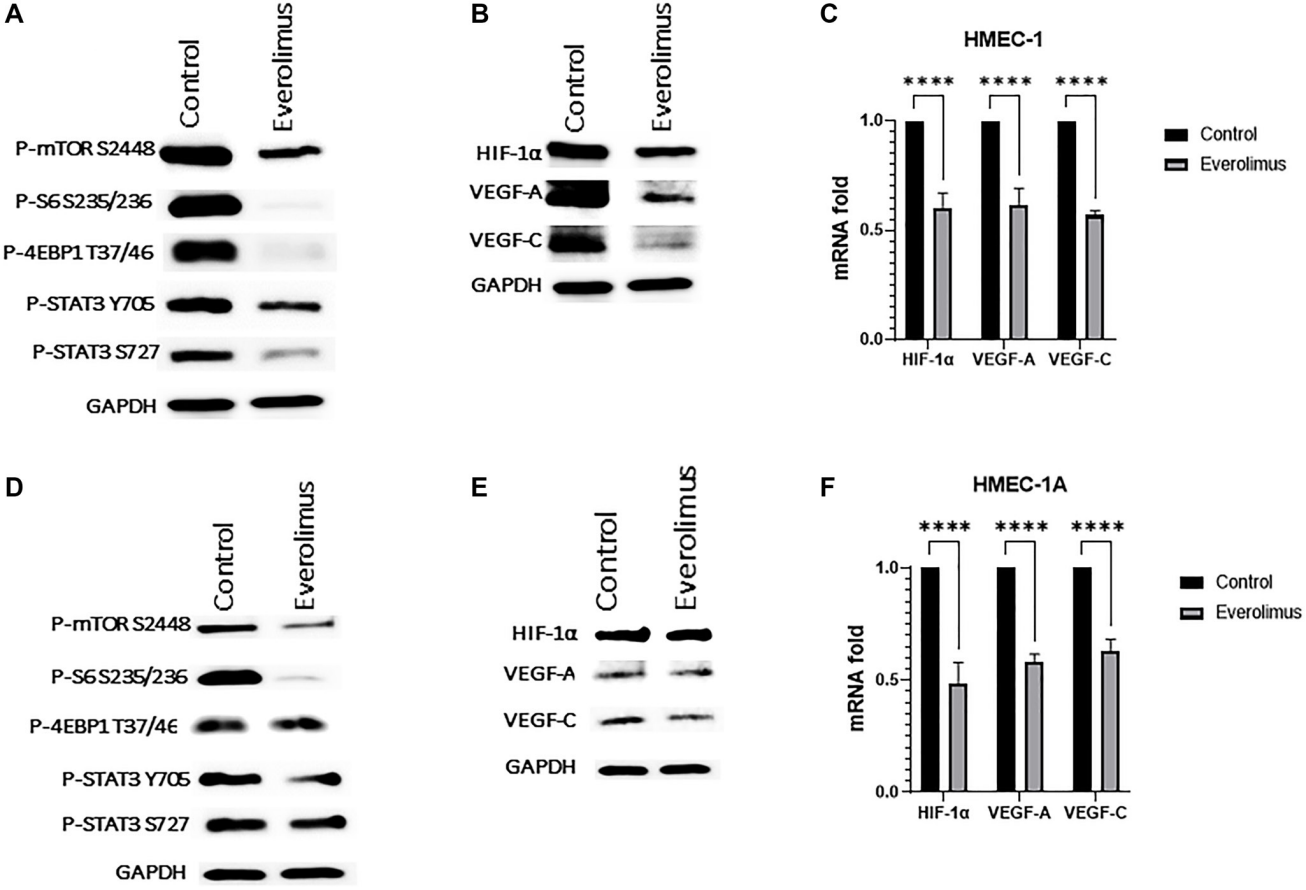
Everolimus inhibits mTORC1/STAT3 pathway and downregulates HIF-1α, VEGF-A and VEGF-C in HMEC-1 and HMEC-1A cells. (**A**) Proteins were prepared from HMEC-1 treated with 100 nm of everolimus for 24 hours. Representative western blot showing inhibition of mTORC1 pathway proteins and STAT3 phosphorylation. (**B**) Representative western blot of HIF-1α, VEGF-A and VEGF-C in HMEC-1 showing downregulation by everolimus treatment. (**C**) Quantification of qRT-PCR data showing mRNA fold change for HIF-1α, VEGF-A and VEGF-C in HMEC-1. ^****^ *P* < 0.00005 vs. control, ANOVA. Data represent the mean +/− SEM of three independent experiments, each comprising samples in triplicate. mRNA levels of all were significantly reduced by everolimus treatment. (**D**) Proteins were prepared from HMEC-1A cells treated with 100 nm of everolimus for 24 hours. Representative western blot showing inhibition of mTORC1 and STAT3 phosphorylation in HMEC-1A. (**E**) Representative western blot of HIF-1α, VEGF-A and VEGF-C in HMEC-1A showing downregulation by everolimus treatment. (**F**) Quantification of qRT-PCR data showing mRNA fold change for HIF-1α, VEGF-A and VEGF-C in HMEC-1A. ^****^ *P* < 0.00005 vs. control, ANOVA. Data represent the mean +/− SEM of three independent experiments, each comprising samples in triplicate. mRNA levels of all were significantly reduced by everolimus treatment.

Angiogenesis involves multiple processes of neovascularization that includes endothelial cell proliferation, migration, and formation of lumen morphogenesis on matrigel. Everolimus significantly inhibited cell proliferation of HMEC-1 when cells were treated with 100 nm for 72 hours (Supplementary Figure 1A). Next, we evaluated *in vitro* tube formation in cells treated with 100 nm everolimus for 16 hours, where everolimus significantly reduced tube morphology and network formation of HMEC-1 cells (Supplementary Figure 1B). The evaluation of everolimus on endothelial cell migration, where cells were treated with 100 nm everolimus for 8 hours, showed a significant reduction in the number of migrated cells (Supplementary Figure 1C). Moreover, P-mTOR S2448, P-S6 S235/236, P-4EBP1 T37/46 and P-STAT3 were downregulated in both HMEC-1 (Figure 5A) and HMEC-1A (Figure 5D) cell lines. Consistent with the HNSCC cell lines, everolimus reduced levels of HIF-1α, VEGF-A and VEGF-C in both HMEC-1 (Figure 5B) and HMEC-1A (Figure 5E) cells. Finally, the mRNA levels of HIF-1α, VEGF-A and VEGF-C were significantly reduced by everolimus treatment in both HMEC-1 (Figure 5C) and HMEC-1A (Figure 5F).

## DISCUSSION

*TP53* mutations are associated with treatment resistance and shorter survival [32, 33]. Therefore, patients with *TP53* mutations often exhibit persistent disease or MRD and potentially could benefit from adjuvant therapy. A multi-institutional phase-2 clinical trial subset analysis determined that HNSCC patients with *TP53* mutations benefited when everolimus was administered as adjuvant therapy [13]. Everolimus inhibits STAT3/HIF-1α/VEGF pathways in wt TP53 cell lines as well (data not shown). However, our goal was not to compare p53 mutated tumors to p53 wt tumors, as patients with p53 wt respond so well and do not require adjuvant therapy. Most trials are aimed at de-escalation of treatment for p53 wt tumor patients to decrease side effects. However, we sought specifically to determine the mechanism of why patients with p53 mutated tumors benefited from mTORi.

We employed *TP53* mutant cell lines and tumor-cell xenografts to investigate the underlying mechanisms of the anti-tumorigenic effects of everolimus. To recapitulate the clinical trial design, we utilized the minimum residual disease (MRD) model using HN31 and FaDu xenografts [34]. MRD is closely associated with disease persistence/recurrence. Therefore, a better understanding of the underlying mechanism and targets related to the MRD mouse model will be essential to effectively preventing the progression of upper aerodigestive tract cancers. Our prior published studies established mTOR inhibitors as possible adjuvant therapy for microscopic residual disease in HNSCC [34]. However, the underlying mechanism for mTORi growth inhibitory effects in MRD model is largely unknown. Treatment with everolimus significantly inhibited the growth of *TP53* mutant cell lines and attenuated the growth kinetics of tumor-cell xenografts. In accordance with our previous studies [35], everolimus inhibited mTORC1 activity by downregulating P-mTOR S2448, P-S6 S235/236, and 4EBP1 T70. Moreover, both in cell lines and xenografts, P-STAT3, HIF-1α, VEGF-A and VEGF-C were downregulated with everolimus treatment. Interestingly, some studies suggest that mTORi do not affect the stability of HIF-1α and act independently of the Von Hippel-Lindau (VHL) protein [36, 37]. Instead, mTORi inhibits the translation of HIF-1α through downregulation of P-4EBP1 and represses transcription of HIF-1α through reduction of P-STAT3 [24]. Our study showed similar results in *TP53* mutant HNSCC cell lines and xenografts. Everolimus downregulated HIF-1α, accompanied by the decrease in P-4EBP1 and P-STAT3. Therefore, in line with other studies we postulate that everolimus-mediated downregulation of HIF-1α involves inhibition of its translation (through downregulation of P-4EBP1) and repression of its transcription (through downregulation of P-STAT3). Since the HIF-1α targets, VEGF-A and VEGF-C, are involved in tumor angiogenesis and lymphangiogenesis, we further assessed the effects of everolimus on potential crosstalk between tumor and vascular endothelial and lymphatic cells. Our results revealed that everolimus significantly inhibited tumor angiogenesis and lymphangiogenesis in HN31 and FaDu xenografts, potentially via a paracrine effect. Tumor cells secrete VEGF-A and VEGF-C in the tumor microenvironment which promotes tumor angiogenesis and lymphangiogenesis. VEGF-A mediated angiogenesis plays a pivotal role in tumor recurrence and growth [25–27], whereas VEGF-C mediated lymphangiogenesis is crucial for invasion and metastasis [28–30]. Therefore, we postulate that the inhibition of tumor angiogenesis and lymphangiogenesis by everolimus prevents the recurrence of *TP53* mutant HNSCC tumors. Finally, we have shown that everolimus substantially reduces cell proliferation, *in vitro* network formation, and migration of the endothelial cell line HMEC-1 with concomitant downregulation of the STAT3/HIF-1α/VEGF pathway.

mTOR is well established as a positive regulator of HIF-1α expression and its transcriptional activity [36–39]. The downregulation of HIF-1α target genes by mTORi prevented the growth of renal cell carcinoma [36]. However, the underlying mechanisms for everolimus-induced inhibitory effect on tumor growth have not been elucidated in *TP53* mutant HNSCC. Considering the molecular context of *TP53* mutant HNSCC, mutant 53 protein causes sustained activation of the mTOR pathway to upregulate HIF-1α, which has been implicated in the expansion of residual cancer stem cells in colorectal cancer [40]. This residual cancer stem cell that survives definitive therapy leads to tumor recurrence [41–44]. Therefore, we postulate that the inhibition of HIF-1α-mediated angiogenesis by everolimus plays a pivotal role in preventing tumor recurrence of *TP53* mutant HNSCC. However, HIF-1α target genes are also involved in other critical aspects of cancer biology, including cell survival, chemotherapy and radiation resistance, immortalization, immune evasion, metastasis, and metabolism. The intervention of these oncological processes might also halt the progression of *TP53* mutant HNSCC, which has not been explored in this current study. Previous work from our lab has shown that everolimus induces autophagy-dependent cell death (ADCD) in *TP53* mutant HNSCC through tumor cell-intrinsic mechanisms [35]. This study demonstrated that everolimus inhibits tumor angiogenesis and lymphangiogenesis through the downregulation of HIF-1α within the tumor microenvironment. Therefore, we conclude that prevention of TP53 mutant HNSCC tumor growth by everolimus consists of multifaceted mechanisms that involve modulation of both tumor cells and the tumor microenvironment. Further studies are required to elucidate the underlying detailed mechanisms.

## MATERIALS AND METHODS

### Cell culture

Three HPV-negative HNSCC cell lines with TP53 mutations were used in this study: 1) HN31 (C176F and A161S), kindly provided by Dr. Jeffrey Myers at The University of Texas M.D. Anderson Cancer Center, 2) FaDu (R248L) procured from American Type Culture Collection, and 3) UPCI-SCC114 (SCC114) (R248Q), kindly provided by Dr. Susanne Gollin at the University of Pittsburgh. Information on TP53 mutational status and disease background of cell lines are described previously [35]. These cell lines have Evolutionary Action (EA) scores of more than 75, therefore considered as high risk *TP53* mutations [45]. All cell lines were maintained in Dulbecco-modified Eagle medium (Corning, Corning, NY, USA), 10% fetal bovine serum (R&D Systems, Minneapolis, MN, USA), antibiotic/antimycotic (Hyclone, Logan, UT, USA), glutamine (Sigma-Aldrich, St. Louis, MO, USA), sodium pyruvate (Sigma-Aldrich, St. Louis, MO) and non-essential amino acids (Sigma-Aldrich, St. Louis, MO, USA). HMEC-1 and HMEC-1A, human endothelial and lymphatic endothelial cell lines, respectively, were kindly provided by Dr. J. Steven Alexander at LSU Health Sciences Center-Shreveport and maintained in MCDB 131 medium (Sigma-Aldrich, St. Louis, MO, USA), supplemented with 20 mM HEPES, 1 ug/ml hydrocortisone, 10 ng/ml EGF and 10% fetal bovine serum. Cells were grown in monolayers and maintained in humidified 5% CO2 atmosphere at 37°C.

### Western blot

Cells were grown in their respective medium and treated with 100 nm of everolimus (Selleckchem, Houston, TX) for 24 hours. The cells were lysed using 1X cell lysis buffer (Cell Signaling Technology, Danvers, MA, USA) containing a Protease Inhibitor Cocktail (Roche Molecular Biochemicals, Germany) and phosphatase inhibitors (Sigma-Aldrich, St. Louis, MO, USA) [46]. Briefly, protein lysates (30–50 μg) were denatured using Laemmli Sample Buffer then loaded, run in precast gels, and transferred to PVDF membranes using the Trans-Blot Turbo Transfer System (Bio-Rad, Hercules, CA, USA). After blocking with either BSA (3%) or milk (5%), membranes were incubated with primary antibody overnight at 4^°^C. The primary antibodies used were HIF-1α, P-mTOR S2448, mTOR, P-S6 S235/236, P-STAT3 Y705, P-STAT3 S727, GAPDH (Cell Signaling Technology, Danvers, MA, USA), VEGF-A, and VEGF-C (Abcam, Cambridge, UK). The membranes were next incubated with either an anti-rabbit HRP-conjugated secondary antibody (R&D Systems, Minneapolis, MN, USA), or an anti-mouse HRP-conjugated secondary antibody (Cell Signaling Technology, Danvers, MA, USA) for one hour. The chemiluminescent signal was developed using SuperSignal Chemiluminescent Substrates (Thermo Fisher Scientific, Waltham, MA, USA), and captured with a ChemiDoc XRS+ System (Bio-Rad, Hercules, CA, USA). Acquired images were analyzed and quantified using Image J software.

### Cell viability assay

HNSCC cell lines were seeded in 96-well tissue culture plates at a density of 1000–2000 cells/well for 24 hours. Cells were then treated with everolimus (10 and 100 nm) for 72 hours. For HMEC cell lines, 8000 cells/well were seeded in a 96-well plate for 24 hours, followed by 72 hours of treatment with everolimus. After respective treatments, cell viability was measured using the CellTiter 96^®^ Aqueous cell proliferation assay according to the manufacturer’s instructions (Promega Corporation, Madison, WI, USA). Cell viability was quantified and expressed as percent control.

### HNSCC xenograft model

The mouse xenograft experiment was conducted in compliance with the Louisiana State University Health Sciences Center Institutional Animal Care and Use Committee guidelines under the U.S. Public Health Service Policy on Humane Care and Use of Laboratory Animals. 1 × 10^6^ FaDu cells or 2 × 10^6^ HN31 cells were injected subcutaneously into both flanks of 6-8 weeks old female athymic nude mice (Charles River Laboratories, Shrewsbury, MA, USA). Tumors were measured using a digital caliper and volume calculated using the formula [(length × width^2^)/2]. Mice were randomized to two groups of 20 mice each. To mimic minimal residual disease in patients, treatment started on day five before the appearance of tumors. The experimental group received 5 mg/kg of everolimus dissolved in 1% CMC-Na (Sigma-Aldrich, St. Louis, MO, USA) by oral gavage daily, whereas control mice received 1% CMC-Na. Tumor volume and body weight were measured three times per week. After three weeks, mice were sacrificed, the tumors excised, and tumor lysates were prepared using RIPA buffer (Cell Signaling Technology, Danvers, MA, USA). Tumor lysates were then analyzed by Western blot.

### Immunohistochemistry and microvascular density quantification

The xenograft tissue was fixed in zinc-formalin and paraffin embedded. 4 μm FFPE tissue sections were deparaffinized, dehydrated, processed with antigen retrieval buffer, and incubated with primary antibodies. For CD31 staining, citrate buffer (pH 6.0), and LYVE-1, EDTA buffer (pH 9.0) was used for antigen retrieval following the manufacturer’s protocol using a pressure cooker. The slides were then incubated with either anti-CD31 (Cell Signaling Technology, Danvers, MA, USA), or anti-LYVE1 (R&D Systems, Minneapolis, MN, USA) antibody for 24 hours at 4^°^C. After incubation with biotinylated secondary antibody (Vector Lab, Burlingame, CA, USA for 30 min, slides were incubated with streptavidin-biotin-peroxidase (Vector Lab, Burlingame, CA, USA). DAB substrate was used to visualize positive staining. Images were acquired with the Olympus VS200 Research Slide Scanner under 40× magnification. Microvascular density was measured using the Aperio Microvessel Analysis Algorithm (Aperio, Vista, CA, USA).

### ELISA

The amount of VEGF-A secreted in the cell culture medium was measured using VEGFA Human ELISA Kit (Abcam, Cambridge, UK) according to the manufacturer’s protocol. Briefly, FaDu, SCC114 and HN31 cell lines were seeded overnight in a 6-well plate and treated with 100nM of everolimus for 24 hours. Cell medium was collected and centrifuged at 500 RCF. 50 ul of supernatant was used to determine VEGF-A levels secreted in the medium.

### Endothelial cell proliferation assay

Exponentially growing HMEC-1 cells were plated at a density of 8 × 10^3^ cells per well in 96-well plates and grown in a complete MCDB131 medium (Sigma-Aldrich, St. Louis, MO, USA) containing 10% FBS for 24 hours. After 24 hours, cells were treated with either 100 nm of everolimus or 100 ng/mL of VEGF (R&D Systems, Minneapolis, MN, USA) or a combination of everolimus and VEGF for 72 hours. Cell viability was measured using the CellTiter 96^®^ Aqueous cell proliferation assay according to the manufacturer’s instructions (Promega, Madison, WI, USA). The cell viability was quantified and expressed as percent control.

### *In vitro* angiogenesis (tube morphology) assay

2.5 × 10^3^ cells in 200 μl of MCDB 131 medium supplemented with 0.5% FBS and either everolimus, VEGF, or both were added to the wells of 96 well plates pre-coated with growth factor–depleted matrigel (7 μg/mL; Becton Dickinson, Bedford, MA, USA). After 16 hours, the medium was removed gently without disturbing newly formed tubules. Images were captured at 40× magnification on a Leica microscope. The image was analyzed using angioanalyzer plugins to measure the number of branch points and segments. Five random 40× images were used for each well. Each experiment was done in triplicate and repeated twice.

### Endothelial cell migration assay

Migration assays were done in transwell tissue culture plates (6.5 mm and 4-μm pore size, Becton Dickinson and Co., Franklin Lakes, NJ, USA). The bottom of the transwell chamber was coated with 10 mg/mL of collagen I (Sigma-Aldrich, St. Louis, MO, USA) for 30 minutes. 50 × 10^3^ cells were seeded into each well and allowed to migrate for 6 hours. The cells were then fixed with methanol and stained with crystal violet. Migrated cells were counted in five random 10× fields. Each experiment was done in triplicate and repeated twice. The results are expressed as mean number and number of cells +/− SE.

## SUPPLEMENTARY MATERIALS



## Abbreviations

HNSCC: Head and Neck Squamous Cell Carcinoma
MRD: Minimal Residual Disease Model
mTORi: mTOR Inhibitor
HMEC: Human Microvascular Endothelial Cells
ELISA: Enzyme Linked Immunosorbent Assay
FFPE: Formalin-Fixed, Paraffin-Embedded
