# Supplementary material for: Everolimus downregulates STAT3/HIF-1α/VEGF pathway to inhibit angiogenesis and lymphangiogenesis in TP53 mutant head and neck squamous cell carcinoma (HNSCC)
Source: Oncotarget. 2023 Feb 2;14:85–95. doi: 10.18632/oncotarget.28355 (PMC9901561; doi:10.18632/oncotarget.28355)
Supplement: Supplementary file 1 [file oncotarget-14-28355-s001.pdf]

Everolimus downregulates STAT3/HIF-1α/VEGF pathway to inhibit angiogenesis and lymphangiogenesis in *TP53* mutant head and neck squamous cell carcinoma (HNSCC)

SUPPLEMENTARY MATERIALS

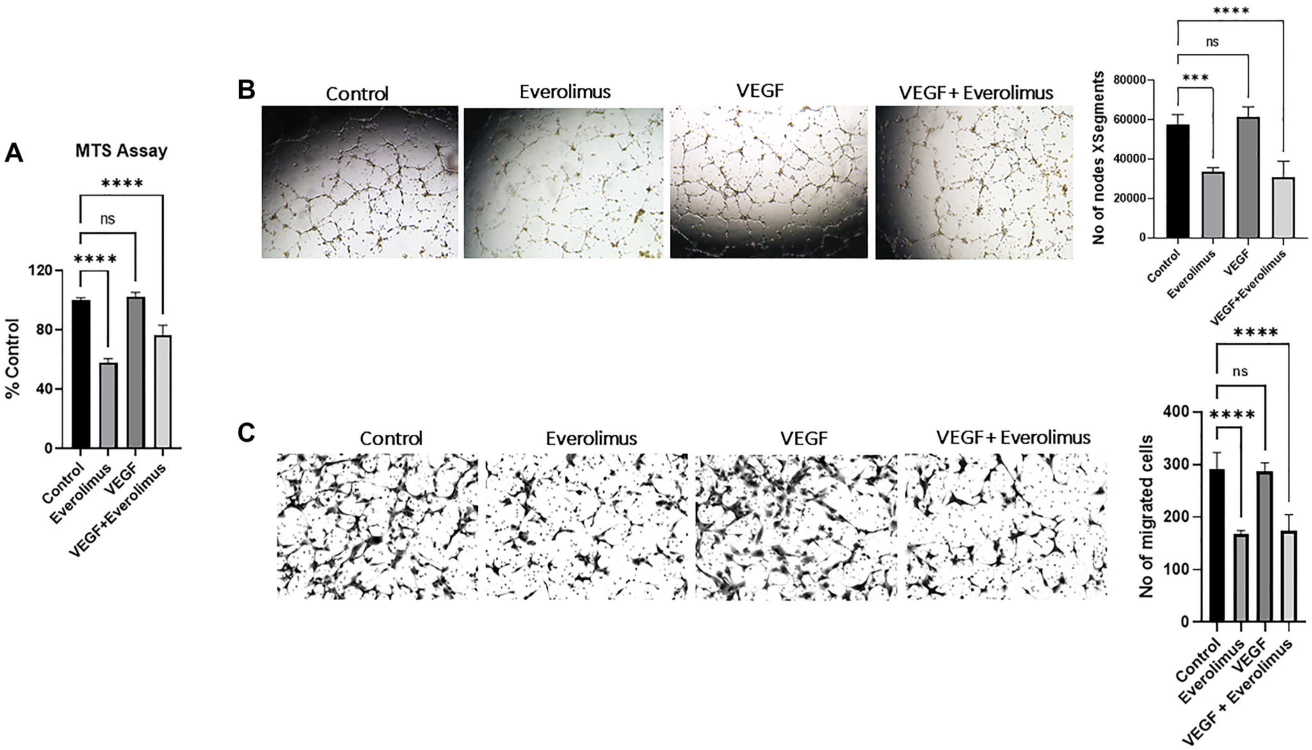

Supplementary Figure 1: Everolimus inhibits proliferation, migration and tube formation of human microvascular cell line (HMEC-1). (**A**) The effect of everolimus on cell viability was measured as a percent of control (untreated cells) everolimus significantly inhibits the proliferation of HMEC-1 cell line. $^{****}P < 0.0005$ vs. control, ANOVA. Data represent the mean +/− SEM of three independent experiments, each experiment comprising samples in triplicate. (**B**) The representative image and quantification of *in vitro* angiogenesis assay. Everolimus significantly reduces the vessel density of HMEC-1 cell line. Columns, mean number of branches per network X number of networks ($n = 12$); bars, +/− SEM. $^{****}P < 0.00005$ (one-way ANOVA). (**C**) Representative image and quantification of HMEC-1 migration assay. Everolimus significantly reduces the number of migrated cells. Columns represents number of migrated cells per 100 field, ($n = 12$); bars, +/− SEM. $^{**}P < 0.005$; $^{***}P < 0.0005$, vs. control (one-way ANOVA). Each experiment was repeated three times.
